# The Okur-Chung Neurodevelopmental Syndrome Mutation CK2^K198R^ Leads to a Rewiring of Kinase Specificity

**DOI:** 10.3389/fmolb.2022.850661

**Published:** 2022-04-19

**Authors:** Danielle M. Caefer, Nhat Q. Phan, Jennifer C. Liddle, Jeremy L. Balsbaugh, Joseph P. O’Shea, Anastasios V. Tzingounis, Daniel Schwartz

**Affiliations:** ^1^ Department of Physiology and Neurobiology, University of Connecticut, Storrs, CT, United States; ^2^ Center for Open Research Resources and Equipment, Proteomics and Metabolomics Facility, University of Connecticut, Storrs, CT, United States

**Keywords:** CK2, Okur-Chung neurodevelopmental syndrome, kinase specificity, proteomics, ProPeL, phosphorylation, mass spectrometry

## Abstract

Okur-Chung Neurodevelopmental Syndrome (OCNDS) is caused by heterozygous mutations to the *CSNK2A1* gene, which encodes the alpha subunit of protein kinase CK2. The most frequently occurring mutation is lysine 198 to arginine (K198R). To investigate the impact of this mutation, we first generated a high-resolution phosphorylation motif of CK2^WT^, including the first characterization of specificity for tyrosine phosphorylation activity. A second high resolution motif representing CK2^K198R^ substrate specificity was also generated. Here we report the impact of the OCNDS associated CK2^K198R^ mutation. Contrary to prior speculation, the mutation does not result in a complete loss of function, but rather shifts the substrate specificity of the kinase. Broadly speaking the mutation leads to 1) a decreased preference for acidic residues in the +1 position, 2) a decreased preference for threonine phosphorylation, 3) an increased preference for tyrosine phosphorylation, and 4) an alteration of the tyrosine phosphorylation specificity motif. To further investigate the result of this mutation we have developed a probability-based scoring method, allowing us to predict shifts in phosphorylation in the K198R mutant relative to the wild type kinase. As an initial step we have applied the methodology to the set of axonally localized ion channels in an effort to uncover potential alterations of the phosphoproteome associated with the OCNDS disease condition.

## Introduction

Okur-Chung Neurodevelopmental Syndrome (OCNDS) is broadly characterized by delayed psychomotor development and intellectual disability (Okur et al., 2016). The disorder has been linked to various heterozygous mutations on the *CSNK2A1* gene, which encodes the alpha subunit of protein kinase CK2, a well-characterized and conserved serine/threonine protein kinase (Pinna, 1990, 2003; Meggio and Pinna, 2003). Of the mutations associated with OCNDS, the mutation of lysine-198 to arginine (K198R) has been most frequently observed. The K198R mutation has been speculated to result in a loss of kinase function (Chiu et al., 2018), and this speculation was more recently thought to be confirmed (Dominguez et al., 2021). Interestingly however, lysine-198 is located in the activation segment of CK2 and had been previously implicated in the acidic residue preference of CK2 at the +1 position relative to the phosphoacceptor (Sarno et al., 1997). Furthermore, Sarno et al. demonstrated that while the K198A mutation reduced phosphorylation of substrates with a +1 acidic residue, it allowed for efficient phosphorylation of substrates with a +1 alanine (Sarno et al., 1996). Taken together, we posited that the CK2^K198R^ mutation may not result in a complete loss of kinase activity, but rather a shift in substrate specificity. To determine the impact on specificity of the CK2^K198R^ mutation we implemented the ProPeL method (Lubner et al., 2018) to generate a high-resolution motif of CK2^WT^ and CK2^K198R^. Additionally, we utilized a probabilistic strategy to determine the differential likelihood of modification for serine, threonine, and tyrosine residues on axonally localized ion channels under wild type and mutant kinase conditions.

## Materials and Methods

### Plasmids

For bacterial expression, a plasmid containing the human CSNK2A1 gene in an Invitrogen Gateway donor vector (pDONR223) was provided by The Broad Institute and was transferred to the pDEST17 backbone following the standard Gateway Protocol (Life Technologies). Generation of the CK2^K198R^ mutant was done using the Q5^®^ Site-Directed Mutagenesis Kit (New England BioLabs).

### ProPeL Experiments for Motif Determination

The Proteomic Peptide Library (ProPeL) approach is a mass spectrometry-based method to experimentally determine high-resolution kinase specificity motifs. In this method, an exogenous kinase of interest is expressed in *Escherichia coli* and phosphorylates the endogenous proteome according to its native substrate preferences. These phosphorylated proteins can then be identified by LC-MS/MS to create a list of phosphorylation sites to be used to visualize substrate specificity. ProPeL experiments were carried out as previously described (Chou et al., 2012; Lubner et al., 2018) with the following conditions for *in vivo* proteome phosphorylation: all CK2 constructs were expressed in *E. coli* OverExpress C43 (DE3) cells (Lucigen) by IPTG induction. Optimal expression conditions were determined to be mid-log induction followed by expression for 24 h at 37°C in TB media (data not shown).

### Western Blotting

Comparable expression of CK2^WT^ and CK2^K198R^ was confirmed by western blotting using the primary antibody Anti-CSNK2A1 antibody (Abcam ab10466) at a 1/5,000 dilution and the secondary antibody IRDye^®^ 680RD Donkey anti-Rabbit IgG at a 1/5,000 dilution.

### Untargeted Protein Identification via Tandem Mass Spectrometry

Peptide samples were subjected to mass analysis using a Thermo Scientific Ultimate 3000 RSLCnano ultra-high performance liquid chromatography (UPLC) system coupled to a high-resolution Thermo Scientific Q Exactive HF mass spectrometer. An aliquot of each peptide preparation in Solvent A (0.1% formic acid in H_2_O) was injected onto a Waters nanoEase M/Z Peptide BEH C18 analytical column (130Å, 1.7 μm, 75 μm × 250 mm) and separated by reversed-phase UPLC using a gradient of 4–30% Solvent B (0.1% formic acid in acetonitrile) over a 100- min gradient at 300 nL/min flow. Peptides were eluted directly into the Q Exactive HF using positive mode nanoflow electrospray ionization and 1.5 kV capillary voltage. MS scan acquisition parameters included 60,000 resolution, 1e6 AGC target, maximum inject time of 60 ms, and a 300–1800 m/z mass range. Data-dependent MS/MS scan acquisition parameters included 15,000 resolution, 1e5 AGC target, maximum ion time of 40 ms, loop count of 15, isolation window of 2.0 m/z, dynamic exclusion window of 30 s, normalized collision energy of 27, and charge exclusion “on” for all unassigned, +1, and >+8 charged species.

Peptides were identified using MaxQuant (v1.6.10.43) and the embedded Andromeda search engine (Cox and Mann, 2008). The raw data was searched against three databases: an in-house-generated protein database consisting of 6xHis-tagged CK2 wildtype and mutant sequences, the complete UniProt *E. coli* reference proteome (identifier UP0000068040, accessed 11 September 2020), and the MaxQuant contaminants database. Variable modifications were oxidation of Met, acetylation of protein N- termini, deamidation of Asn/Gln, and for enriched samples, phosphorylation of Ser/Thr/Tyr. Carbamidomethylation of Cys was set as a fixed modification. Protease specificity was set to trypsin, allowing a maximum of two missed cleavages. LFQ quantification was enabled. All results were filtered to a 1% false discovery rate at the peptide spectrum match and protein levels; all other parameters were kept at default values. MaxQuant-derived output was further analyzed in accordance with the ProPeL method (Chou et al., 2012).

### Probability-Based Prediction of CK2^WT^ and CK2^K198R^ Phosphorylation Sites

**FIGURE 1:**
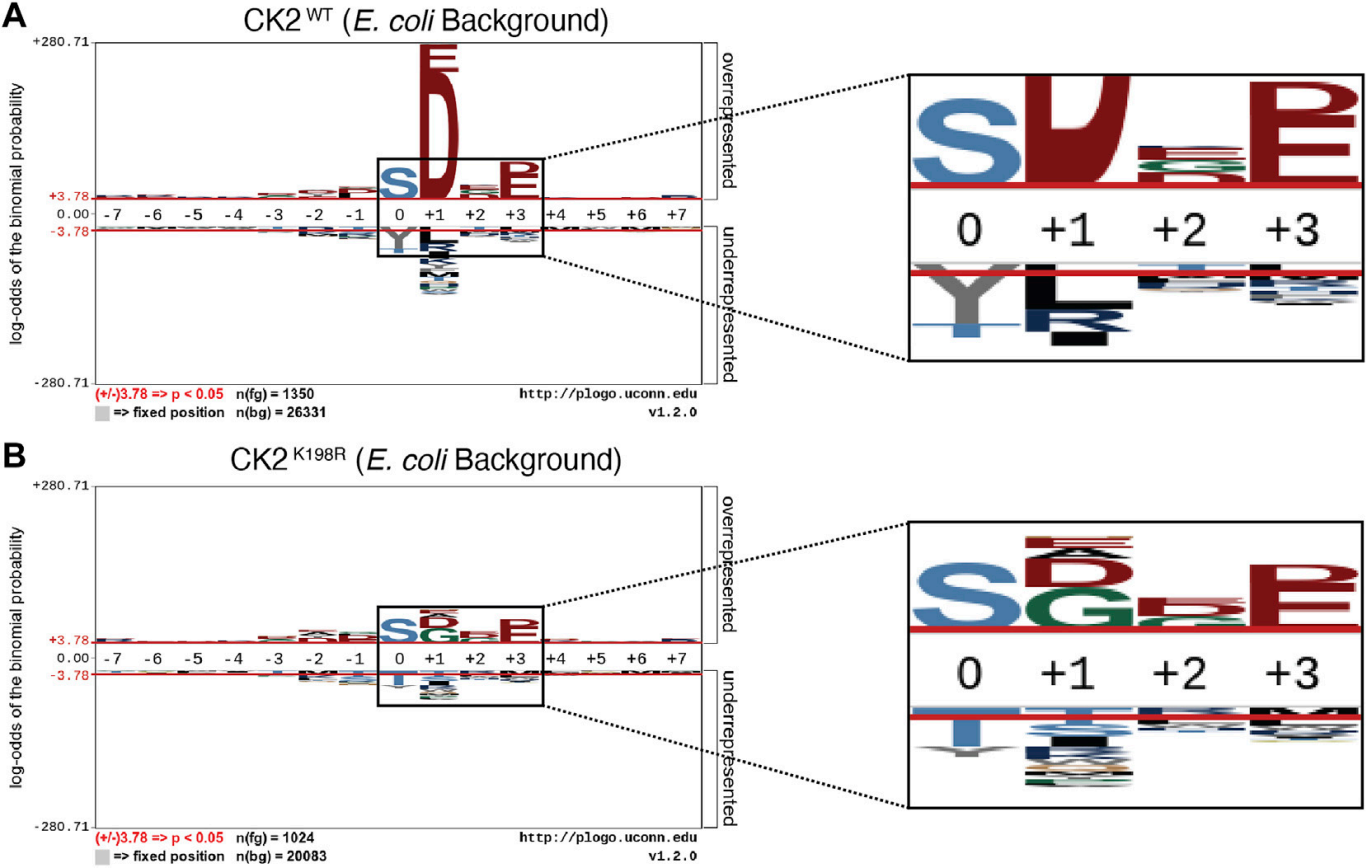
*CK2^K198R^ mutation shifts overall specificity motif*. Substrate specificity of CK2^WT^ **(A)** and CK2^K198R^ **(B)**. The CK2 ^K198R^ mutation results in a decreased preference for acidic (D/E) residues in the +1 position as well as a reordering of phosphoacceptor preference. In CK2^K198R^, a decreased preference for threonine is paired with an increase in preference for tyrosine.

Given that the ProPeL methodology for kinase motif determination is carried out in the context of a closed system (the *E. coli* expressed proteome) the scoring of phosphorylated and unphosphorylated serine, threonine, and tyrosine residues in *E. coli* could be used to convert motif scores to probabilities of modification. Specifically, phosphorylated 15mers (for either the wild type or K198R data set) were mapped back onto the UniProt reference K12 *E. coli* proteome to generate a list of expressed proteins phosphorylated by CK2 in *E. coli.* These proteins were then subjected to a full trypsin digestion *in silico* and resultant peptides with a length up to 50 residues were retained to generate a list of all potential phosphorylation sites that could have been hypothetically observed in our experiments. Seven residues upstream and downstream from each serine, threonine, and tyrosine residue in these peptides was extracted to create a complete list of serine, threonine, and tyrosine-centered 15mers. Each 15mer was labeled as either positive or negative depending on whether it was observed to be phosphorylated in our ProPeL experiments, resulting in 1,350 positive (phosphorylated) and 26,331 negative (not observed to be phosphorylated) 15mers in the CK2^WT^ data set, and 1,024 positive and 20,083 negative 15mers in the CK2^K198R^ data set. Every peptide was scored using the appropriate wild type or K198R serine, threonine, or tyrosine-centered position weight matrix derived from the log-odds binomial probabilities observed in the corresponding serine, threonine, or tyrosine-centered pLogo, as we have shown previously (Schwartz et al., 2009). Note, the value for the central phosphorylated residue was derived from pLogo containing phosphorylated serine, threonine, and tyrosine-centered 15mers relative to presumed unphosphorylated serine, threonine, and tyrosine-centered 15mers (i.e., the pLogo with the unfixed central residue, see Figure 1). The complete set of scored 15mers was sorted in descending order and was used as a reference table to determine probabilities of modification based on score - specifically by taking the number of positive peptides above a score threshold divided by the total number of positive and negative peptides above a score threshold. Complete reference tables for CK2^WT^ and CK2^K198R^ are available in Supplementary Tables S5, S6. This methodology was utilized to score and assign probabilities of modification to all serine, threonine, and tyrosine residues in human axonally localized ion channels (see Table 1, Supplementary Table S7).

**TABLE 1:** Top ten phosphorylation sites predicted to be most differentially phosphorylated by CK2^WT^ and CK2^K198R^ in axonally localized human ion channels.

Protein name	Position	Sequence	CK2^WT^ probability (%)	CK2^K198R^ probability (%)	Probability difference	Known phosphorylation site
SCN1A	510	KRKQKEQS^*^GGEEKDE	34	100	66%	Paralog to known SCN2A S514 site
SCN2A	514	KKKQKEQS^*^GEEEKND	35	100	65%	^b^Yes, Human
KCNA1	438	VSSPNLAS^*^DSDLSRR	64	23	−40%	^b^Yes, Human
SCN2A	1,126	TEEFSSES^*^DMEESKE	83	44	−39%	^a^Yes, Human
CACNA1H	1,027	AEGDANRS^*^DTDEDKT	64	25	−39%	No
CACNA1D	1,266	FKPKGYFS^*^DAWNTFD	61	23	−39%	No
SCN1A	1,136	TEDFSSES^*^DLEESKE	83	44	−38%	Paralog to known SCN2A S1126 site
CACNA1H	2,094	GGEEAEAS^*^DPADEEV	61	24	−37%	No
CACNA1D	2,138	QDFGPGYS^*^DEEPDPG	81	44	−37%	No
KCNQ2	839	PYIAEGES^*^DTDSDLC	58	22	−36%	^b^Yes, Mouse

a Sites were considered to be known if they appeared on PhosphoSitePlus.

b Sites were identified in fewer than three high throughput experiments.

## Results

### CK2^K198R^ is an Active Kinase

To obtain a higher resolution motif than we previously published in 2012 (Chou et al., 2012) for CK2^WT^, several new ProPeL experiments were carried out utilizing more sensitive instrumentation that allowed confident identification of low abundance phosphorylated peptides that escaped identification previously. Experiments with CK2^WT^ identified 3,894 tryptic phosphopeptides (Supplementary Table S1) with 2,845 phosphopeptides classified as high confidence (i.e., containing at least one phosphorylation site with greater than 0.9 probability of localization determined by MaxQuant/Andromeda). Parallel ProPeL experiments with CK2^K198R^ identified 3,322 (Supplementary Table S2) tryptic phosphopeptides with 2,439 phosphopeptides classified as high confidence. Each high confidence phosphorylation site was mapped back to the UniProt reference K12 *E. coli* proteome and extended to create lists of unique 15mers as previously described (Lubner et al., 2018). Finally, 312 phosphorylation sites detected by our group and others as endogenous to *E. coli* (Macek et al., 2008; Chou et al., 2012; Soares et al., 2013; Lubner et al., 2016) were removed from each list prior to motif analysis. This process yielded a data set comprised of 1,350 unique phosphorylation sites (818 pS, 422 pT, 110 pY) for CK2^WT^ (Supplementary Table S3) and 1,024 unique phosphorylation sites (619 pS, 251 pT, 154 pY) for CK2^K198R^ (Supplementary Table S4). Taken together these results indicate that CK2^K198R^ is an active kinase able to phosphorylate substrates under *in vivo* conditions. Furthermore, given that the CK2^WT^ and CK2^K198R^ ProPeL experiments were carried out under identical conditions, and western blotting revealed comparable levels of protein expression (Supplementary Figure S1), the number of non-unique phosphorylation sites identified could be used as a proxy for kinase activity. We identified 5,362 non-unique phosphorylation sites attributed to CK2^WT^ and 4,146 non-unique phosphorylation sites attributed to CK2^K198R^. When considering the total amount of material analyzed by LC-MS/MS for each kinase (1570 ng for CK2^WT^, 1430 ng for CK2^K198R^) the overall kinase activity of CK2^K198R^ can be approximated at 85% of CK2^WT^, thus suggesting that the OCNDS CK2^K198R^ mutation results in only a slight decrease in kinase activity relative to wild type CK2. This observed activity of CK2^K198R^ stands in contrast to previous results indicating that the mutation results in a loss of activity (Dominguez et al., 2021).

### CK2^K198R^ Exhibits an Altered Substrate Specificity

The generation of pLogos (O’Shea et al., 2013) provides a visual representation of the relative statistical significance of amino acids (represented by single letter abbreviations) at positions within a 15-residue window of a central phosphoacceptor residue. Amino acid preferences at each position are stacked and sized according to significance relative to the background data set. The most significant residues are displayed with the largest size and are positioned closest to the *x*-axis. Background data sets representing unphosphorylated residues were created independently for CK2^WT^ and CK2^K198R^. Each background was generated through the *in silico* tryptic digestion of proteins identified by the presence of at least one phosphorylated peptide in either the CK2^WT^ or CK2^K198R^ experiments. Background lists included serine, threonine, and tyrosine sites in proteins known to be expressed that could potentially have been observed *via* MS/MS but were never detected in a phosphorylated state. These sites were subsequently extended seven amino acids upstream and downstream of each phosphorylatable residue to generate two unique background lists for use in pLogo generation.

The overall pLogo generated with the current CK2^WT^ data set (Figure 1A) recapitulated our previously published CK2^WT^ pLogo (Chou et al., 2012); namely a strong preference for acidic residues (D/E) at the +1 and +3 positions with the +1 position being the most statistically significant position in the pLogo overall. Consistent with our prior hypothesis that the K198R mutation would impact the specificity of the +1 position, the pLogo generated for CK2^K198R^ indicated a massively decreased preference for acidic (D/E) residues in the +1 position, as well as an increased preference for both glycine and alanine, with glycine superseding aspartate to become the most statistically significant residue at the +1 position (Figure 1B). Interestingly, the reduction in significance was limited to the +1 position, as the preference for acidic residues at the +3 position was maintained at nearly identical levels (compare Figures 1A,B).

Further comparison of the CK2^WT^ and CK2^K198R^ derived pLogos indicated an unexpected reordering of phosphoacceptor preference. Though both mutant and wild type CK2 strongly favored serine phosphorylation over threonine or tyrosine phosphorylation, CK2^WT^ strongly disfavored tyrosine as a phosphoacceptor and was relatively neutral towards threonine as a phosphoacceptor (Figure 1A), while CK2^K198R^ strongly disfavored threonine as a phosphoacceptor and was relatively neutral towards tyrosine as a phosphoacceptor. It is important to note that although CK2^K198R^ did not display a preference for tyrosine phosphorylation (i.e., it was not phosphorylated by CK2^K198R^ at a level greater than its relative frequency among phosphorylatable residues), its shift is significant as it represents a near doubling of the proportion of phosphorylated tyrosine residues in the K198R data set (15%) relative to wild type (8.1%).

### CK2^K198R^ Exhibits Altered Substrate Specificity at the Phosphoacceptor Level

**FIGURE 2:**
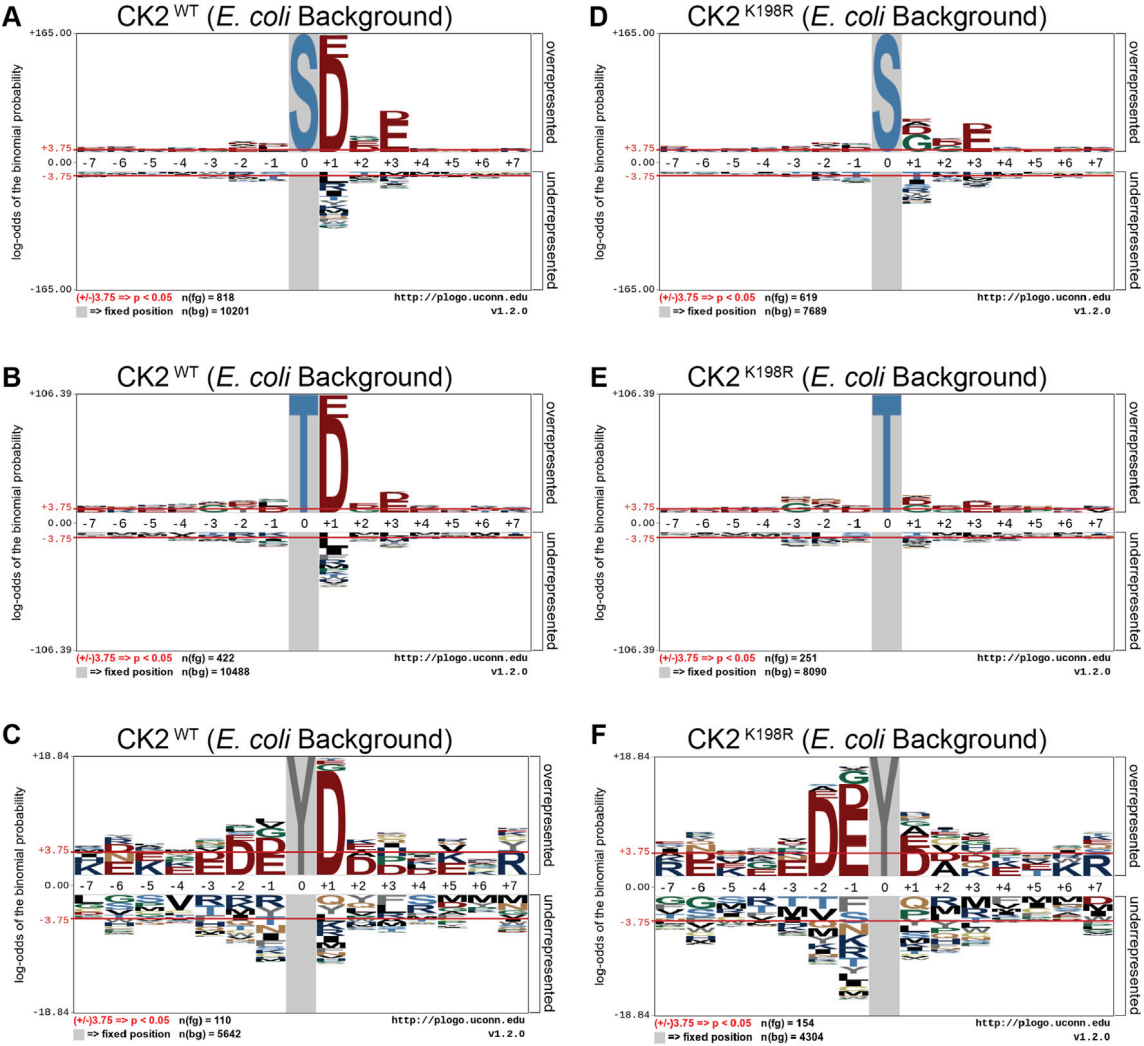
*CK2 ^K198R^ exhibits altered +1 substrate specificity*. Substrate specificity of CK2^WT^ **(A–C)** and CK2^K198R^ **(D–F)**. The CK2 ^K198R^ mutation results in a decreased preference for acidic (D/E) residues in the +1 position for S and T centered phosphorylation sites. For Y centered phosphorylation sites, the preference for acidic residues is shifted to the –2 and –1 positions.

We next investigated whether the decreased preference for acidic residues at the +1 position observed in the overall CK2^K198R^ pLogo was consistent among each individual phosphoacceptor. To investigate this question, we fixed each central residue (serine, threonine, and tyrosine) to generate phosphoacceptor specific pLogos as shown in Figure 2. As was observed in the overall pLogo, the serine and threonine centered CK2^WT^ pLogos indicated a preference for acidic residues at the +1 and +3 positions (Figures 2A,B). The preference for acidic residues was only significant at the +1 position of the tyrosine centered pLogo (Figure 2C). In the CK2^K198R^ serine, threonine, and tyrosine centered pLogos (Figures 2D–F), the decreased preference for acidic residues at the +1 position was preserved. Interestingly however, the tyrosine centered pLogo suggested a modest increased preference for acidic residues at the –1 and –2 positions (Figure 2F). Importantly, the pLogos shown in Figures 2C,F represent the first-ever documentation of a tyrosine motif specificity for CK2.

### CK2^K198R^ Mutation Substantially Alters the Landscape of CK2 Phosphorylation at the Site Level

**FIGURE 3:**
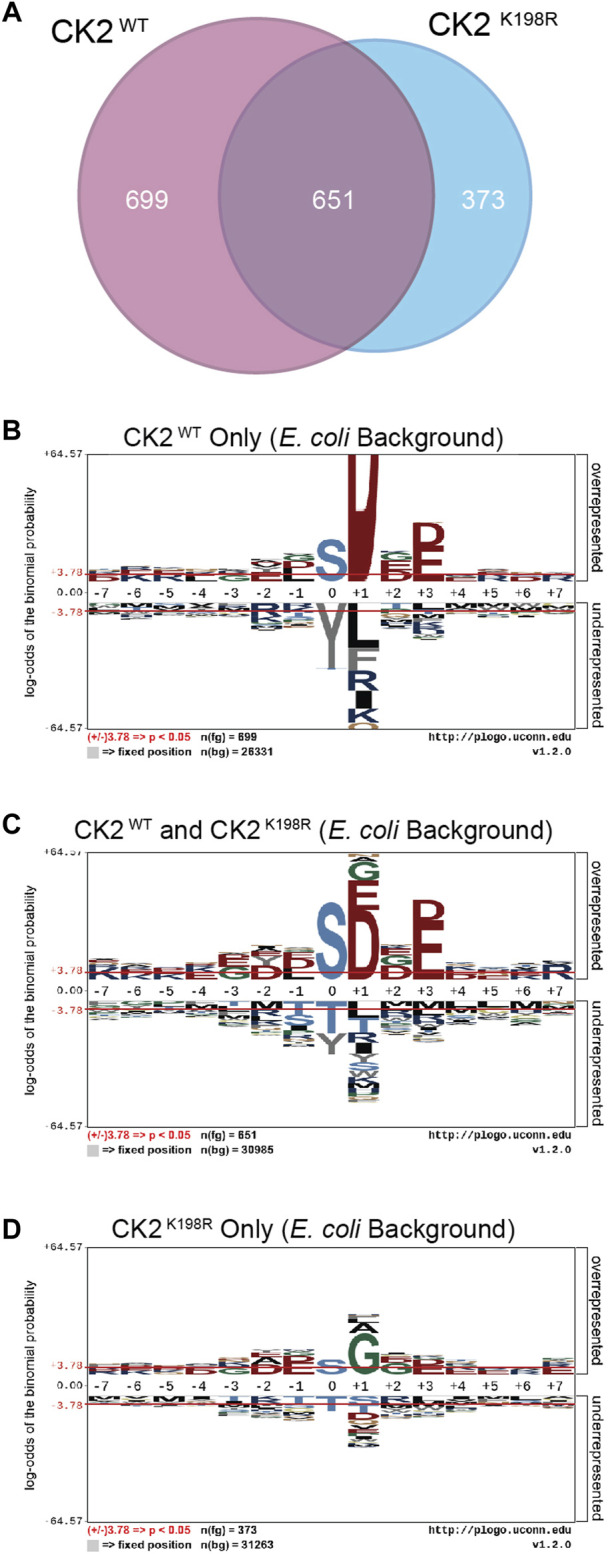
*CK2* ^*K198R*^ *phosphorylates an expanded complement of substrates*. Venn diagram representing the overlap of phosphorylation sites identified in CK2^WT^ and CK2^K198R^ ProPeL experiments **(A)**. Motifs of phosphopeptides identified only in CK2^WT^ **(B)**, both CK2^WT^ and CK2^K198R^ **(C)** or only in CK2^K198R^ **(D)** ProPeL experiments. Phosphopeptides identified in both CK2^WT^ and CK2^K198R^ experiments tend to reflect the canonical CK2^WT^ motif. Those identified only in CK2^K198R^ experiments have a much wider complement of amino acids in the +1 position, indicating increased promiscuity in this position. The phosphoacceptor abundance is also altered, reflecting an increased preference for tyrosine in the K198R mutant.

The ProPeL methodology, carried out in living *E. coli*, provided a unique opportunity to explore the gain/loss of CK2^WT^ and CK2^K198R^ substrates at the site level. Specifically, we were interested in investigating whether our observed decreased preference for acidic residues at the +1 position in CK2^K198R^ resulted from the loss of existing *E. coli* substrates, the addition of completely new substrates, or a combination of the two. Figure 3A shows a Venn diagram representing phosphorylation site overlap between CK2^WT^ and CK2^K198R^ indicating the latter; namely, approximately half of the CK2^WT^ sites were detected in the CK2^K198R^ mutant, yet over a third of the sites detected in the K198R mutant were not observed in the wild type kinase data set.

To further explore motif differences between the three subsets noted in the Venn diagram, pLogos were generated for each subset (Figures 3B–D). The pLogo generated from the set of phosphorylation sites unique to CK2^WT^ included a highly pronounced +1 aspartate specificity that became more attenuated in the overlapping data set and was entirely replaced by glycine (and to a lesser extent, alanine and leucine) in the sites unique to CK2^K198R^. The +3 acidic residues were similarly attenuated in the mutant kinase data set. Finally, with regard to the phosphoacceptor residue, the phosphorylation sites unique to CK2^WT^ showed a highly significant disfavoring of tyrosine residues relative to serine residues that was lost in the CK2^K198R^ mutant (which displayed a general neutrality to the phosphoacceptor residue).

Taken together these results point to a significant shift at the substrate level for the K198R mutant kinase, whereby the most canonical wild type CK2 phosphorylation sites are either lost or phosphorylated with decreased efficiency, coupled with a large increase in previously unphosphorylated sites.

### Predictions of CK2^WT^ and CK2^K198R^ Phosphorylation Sites

CK2^K198R^ is the most frequently occurring mutation in patients with OCNDS (Chiu et al., 2018; Owen et al., 2018). Substrate rewiring in this mutant, indicated by our specificity and *E. coli* substrate analyses, is likely to contribute to the etiology of OCNDS. In an effort to offer a potential mechanism for the role of CK2^K198R^ in OCNDS pathology we developed a predictive strategy for determining CK2^WT^ and CK2^K198R^ phosphorylation sites on human proteins based on our prior *scan-x* post-translational modification predictor (Schwartz et al., 2009), but with the advantage of calculated probabilities of modification (see Methods). Due to the neurodevelopmental nature of OCNDS, in the present study we focused our predictions of potential CK2^WT^ and CK2^K198R^ phosphorylation sites to ion channels localized to the axon. The top ten predicted sites (from a list of over 3,000 total sites) with the greatest change in probability between CK2^WT^ and CK2^K198R^ are listed in Table 1 (a complete list of predicted sites is available in Supplementary Table S7). Of the sites listed, two have an increased probability of modification by CK2^K198R^ and the remaining eight are less likely to be phosphorylated by CK2^K198R^. Additionally, four of the ten sites included in Table 1 have been previously shown to be phosphorylated (with two additional sites being paralogs of phosphorylation sites). We have created a website Located at **https://okurchung.com** that provides users with an interactive view of our predictive results.

## Discussion

Contrary to prior speculation and findings, it is clear from our experiments that the CK2^K198R^ mutation does not simply cause a loss of function, or an inability to recognize substrates (Chiu et al., 2018; Dominguez et al., 2021). We demonstrate that the K198R mutation results in a substantial shift in specificity by reducing the overall preference for acidic (D/E) residues at the +1 position, and by increasing preference for glycine residues at the same position. Differences from prior results (Dominguez et al., 2021) can be attributed to the approach used. In Dominguez et al. the authors utilized a single peptide, RRRADDSDDDDD, to assay the activity of CK2 and its mutant form. Though the peptide would be readily phosphorylated by CK2^WT^, our results predict significantly reduced phosphorylation by CK2^K198R^. Our findings highlight the limitations of individual peptide based approaches to assessing kinase activity, and the importance of implementing unbiased methods such as ProPeL in the study of kinase mutations to gain a more complete understanding of their impact.

While wild type CK2 has previously been shown to be capable of phosphorylating tyrosine residues (Marin et al., 1999; Vilk et al., 2008; Basnet et al., 2014) the study also marks the first time a tyrosine phosphorylation motif has been identified for CK2, and, to our knowledge, the first time that a tyrosine phosphorylation motif has been identified for any kinase previously characterized as primarily phosphorylating serine and threonine residues. Interestingly, our study also revealed a highly significant shift in phosphoacceptor preference, with tyrosine residues being shifted from a disfavored state in CK2^WT^ to a neutral state in the CK2^K198R^ mutant, while threonine residues move in the precise opposite direction. Though the detection of a tyrosine phosphorylation motif for a kinase that disfavors tyrosine phosphoacceptors may appear to be a contradiction, it is important to note that the favoring and/or disfavoring of residues is calculated in the context of the background frequency of those residues. Thus, a phosphoacceptor may still experience significant phosphorylation, while being at a level far below what one would expect due to chance if all phosphoacceptors were phosphorylated at their expected background frequencies. Finally, our work highlights the importance of assessing phosphoacceptor specificity independently, as the shift in the specificity motif from CK2^WT^ to CK2^K198R^ differed between serine/threonine (both of which exhibited similar changes) and tyrosine, which added additional preferences for acidic residues upstream of the modification site in the mutant.

Among the advantages of our ProPeL methodology in assessing kinase specificity over alternate approaches is the fact that the method is carried out under physiological conditions in *E. coli*, thus allowing researchers to assess shifts in phosphorylation at the site level between kinases bearing different characteristics and/or mutations. Utilizing this strategy here we have shown that there exists overlap between CK2^WT^ to CK2^K198R^ at the substrate level, as well as considerable extension to new atypical CK2 substrates by the mutant kinase. Another advantage of ProPeL in the present study is that the closed and finite system (i.e., living bacterial proteomes) allowed us to readily associate *scan-x* phosphorylation scores (Schwartz et al., 2009) with actual probabilities of modification based on the observed modification state of all phosphorylatable residues in *E. coli*. Thus, for any score obtained for any peptide sequence, we could utilize the *E. coli* expressed proteome as a lookup table to correlate scores with probabilities. It is important to note that probabilities obtained utilizing this strategy represent an approximate lower bound on the probability as it is likely that many phosphorylatable sites in the *E. coli* lookup table are considered “negative” when they are indeed phosphorylated, while the converse is unlikely to be true due to our use of conservative peptide identification thresholds (i.e., “absence of proof is not proof of absence”). Most kinases have multiple substrates throughout the cell, only a few of which will directly relate to a phenotype of interest. To investigate signaling changes that may underlie neurodevelopmental phenotypes associated with OCNDS, we focused on neuronal signaling in axonally localized ion channels, as CK2 has been shown to be highly enriched in the axon (Bréchet et al., 2008) and is known to interact with axonal sodium and potassium channels (Hien et al., 2014; Kang et al., 2014; Xu and Cooper, 2015). Further, these ion channels are found to be frequently mutated in both epilepsies and neurodevelopmental disorders (Allen et al., 2020; Malerba et al., 2020), with one recent study reporting 5% of 8,565 participants carrying a mutation in axonally localized voltage gated sodium channels (Lindy et al., 2018; Brunklaus and Lal, 2020). Of 3,000+ sites predicted be differentially phosphorylated by CK2^WT^ and CK2^K198R^, the ten sites with the greatest differences present several interesting candidates for hypothesis generation. One site of particular interest is S839 on KCNQ2, as this site falls in the ankyrin-G (AnkG) binding motif, and is a known CK2 phosphorylation site (Xu and Cooper, 2015). Our predictions indicate that CK2^K198R^ is 36% less likely to phosphorylate S839 than CK2^WT^. Phosphorylation of S839 by CK2 is essential for the accumulation of KCNQ2 in the axon through interaction with AnkG (Kang et al., 2014; Xu and Cooper, 2015) and a loss of this accumulation could be relevant to the pathology of OCNDS patients possessing the CK2^K198R^ mutation. Additional investigation of the interaction between CK2^K198R^ and KCNQ2 is clearly warranted. Though we have produced a website at **https://okurchung.com** that highlights our predictive results on axonally localized ion channels, it is our intention to eventually make publicly available a global proteomic CK2^K198R^ versus CK2^WT^ phosphorylation predictor, as the underlying mechanism by which OCNDS leads to disease is almost certain to be multifactorial given the phenotype is not limited to the central nervous system (Okur et al., 2016; Chiu et al., 2018; Owen et al., 2018).

It is worth noting that while the work presented here demonstrates the impact of the CK2^K198R^ mutation, the most frequently observed mutation in OCDNS patients, to date more than 20 additional OCNDS-associated mutations identified in patients have been described in literature, including both splice variants (Okur et al., 2016; Colavito et al., 2018) and nonsense variants (Nakashima et al., 2019), many of which likely result in a reduction of functional CK2 protein. Though our results present evidence that the CK2^K198R^ mutation results in an active kinase, there is still a potential shared mechanism amongst these variants. Both loss-of-function CK2 mutations and the rewiring of substrate specificity caused by the CK2^K198R^ mutation could lead to the reduced phosphorylation of essential CK2 phosphorylation sites (particularly those where threonine is the phosphoacceptor or an acidic (D/E) residue is present in the +1 position). The additional effects of the CK2^K198R^ mutation, namely increased tyrosine phosphorylation and increased promiscuity towards the +1 position, could contribute in part to the heterogeneity observed in symptoms of OCNDS patients.

Finally, as our group and others previously demonstrated that the Cushing’s Syndrome mutation PKA^L205R^ results in altered substrate specificity (Lubner et al., 2017; Bathon et al., 2019; Walker et al., 2019), the present study provides another data point to suggest that there exists a subset of disease-linked kinase mutations whose deleterious effects can be traced to subtle shifts in substrate specificity rather than broad alterations of kinase activity. The genomic revolution is likely to reveal that we are only at the tip of the proverbial iceberg, as patients with novel mutations are more frequently being identified. By coupling simple experimental strategies for kinase specificity determination with computational approaches to predict substrates, our hope is to generate testable hypotheses regarding underlying mechanisms of disease that can lead us to potential therapeutics faster than ever before.

## Data Availability

The datasets presented in this study can be found in online repositories. The mass spectrometry proteomics data have been deposited to the ProteomeXchange Consortium (http://proteomecentral.proteomexchange.org) via the PRIDE partner repository (Perez-Riverol et al., 2019) with the dataset identifier PXD030823.

## References

[B1] Allen N. M. Weckhuysen S. Gorman K. King M. D. Lerche H. (2020). Genetic Potassium Channel-Associated Epilepsies: Clinical Review of the Kv Family. Eur. J. Paediatric Neurol. 24, 105–116. 10.1016/j.ejpn.2019.12.002 31932120

[B2] Basnet H. Su X. B. Tan Y. Meisenhelder J. Merkurjev D. Ohgi K. A. (2014). Tyrosine Phosphorylation of Histone H2A by CK2 Regulates Transcriptional Elongation. Nature 516, 267–271. 10.1038/nature13736 25252977 PMC4461219

[B3] Bathon K. Weigand I. Vanselow J. T. Ronchi C. L. Sbiera S. Schlosser A. (2019). Alterations in Protein Kinase A Substrate Specificity as a Potential Cause of Cushing Syndrome. Endocrinology 160, 447–459. 10.1210/en.2018-00775 30615103

[B4] Bréchet A. Fache M.-P. Brachet A. Ferracci G. Baude A. Irondelle M. (2008). Protein Kinase CK2 Contributes to the Organization of Sodium Channels in Axonal Membranes by Regulating Their Interactions with Ankyrin G. J. Cel Biol. 183, 1101–1114. 10.1083/jcb.200805169 PMC2600743 19064667

[B5] Brunklaus A. Lal D. (2020). Sodium Channel Epilepsies and Neurodevelopmental Disorders: from Disease Mechanisms to Clinical Application. Dev. Med. Child. Neurol. 62, 784–792. 10.1111/dmcn.14519 32227486

[B6] Caefer D. M. Phan N. Q. Liddle J. C. Balsbaugh J. L. O’Shea J. P. Tzingounis A. V. (2021). The Okur-Chung Neurodevelopmental Syndrome (OCNDS) Mutation CK2K198R Leads to a Rewiring of Kinase Specificity. bioRxiv. 10.1101/2021.04.05.438522 PMC9062000 35517865

[B7] Chiu A. T. G. Pei S. L. C. Mak C. C. Y. Leung G. K. C. Yu M. H. C. Lee S. L. (2018). Okur-Chung Neurodevelopmental Syndrome: Eight Additional Cases with Implications on Phenotype and Genotype Expansion. Clin. Genet. 93, 880–890. 10.1111/cge.13196 29240241

[B8] Chou M. F. Prisic S. Lubner J. M. Church G. M. Husson R. N. Schwartz D. (2012). Using Bacteria to Determine Protein Kinase Specificity and Predict Target Substrates. PLOS ONE 7, e52747. 10.1371/journal.pone.0052747 23300758 PMC3530509

[B9] Colavito D. Del Giudice E. Ceccato C. Dalle Carbonare M. Leon A. Suppiej A. (2018). Are CSNK2A1 Gene Mutations Associated with Retinal Dystrophy? Report of a Patient Carrier of a Novel De Novo Splice Site Mutation. J. Hum. Genet. 63, 779–781. 10.1038/s10038-018-0434-y 29568000

[B10] Cox J. Mann M. (2008). MaxQuant Enables High Peptide Identification Rates, Individualized p.p.b.-range Mass Accuracies and Proteome-wide Protein Quantification. Nat. Biotechnol. 26, 1367–1372. 10.1038/nbt.1511 19029910

[B11] Dominguez I. Cruz-Gamero J. M. Corasolla V. Dacher N. Rangasamy S. Urbani A. (2021). Okur-Chung Neurodevelopmental Syndrome-Linked CK2α Variants Have Reduced Kinase Activity. Hum. Genet. 140, 1077–1096. 10.1007/s00439-021-02280-5 33944995

[B12] Hien Y. E. Montersino A. Castets F. Leterrier C. Filhol O. Vacher H. (2014). CK2 Accumulation at the Axon Initial Segment Depends on Sodium Channel Nav1. FEBS Lett. 588, 3403–3408. 10.1016/j.febslet.2014.07.032 25109776

[B13] Kang S. Xu M. Cooper E. C. Hoshi N. (2014). Channel-anchored Protein Kinase CK2 and Protein Phosphatase 1 Reciprocally Regulate KCNQ2-Containing M-Channels via Phosphorylation of Calmodulin. J. Biol. Chem. 289, 11536–11544. 10.1074/jbc.M113.528497 24627475 PMC4036288

[B14] Lindy A. S. Stosser M. B. Butler E. Downtain‐Pickersgill C. Shanmugham A. Retterer K. (2018). Diagnostic Outcomes for Genetic Testing of 70 Genes in 8565 Patients with Epilepsy and Neurodevelopmental Disorders. Epilepsia 59, 1062–1071. 10.1111/epi.14074 29655203

[B15] Lubner J. M. Balsbaugh J. L. Church G. M. Chou M. F. Schwartz D. (2018). Characterizing Protein Kinase Substrate Specificity Using the Proteomic Peptide Library (ProPeL) Approach. Curr. Protoc. Chem. Biol. 10, e38. 10.1002/cpch.38 29927115 PMC6014603

[B16] Lubner J. M. Church G. M. Chou M. F. Schwartz D. (2016). Reprogramming Protein Kinase Substrate Specificity through Synthetic Mutations. bioRxiv, 091892. 10.1101/091892

[B17] Lubner J. M. Dodge‐Kafka K. L. Carlson C. R. Church G. M. Chou M. F. Schwartz D. (2017). Cushing's Syndrome Mutant PKA L 205R Exhibits Altered Substrate Specificity. FEBS Lett. 591, 459–467. 10.1002/1873-3468.12562 28100013 PMC5321106

[B18] Macek B. Gnad F. Soufi B. Kumar C. Olsen J. V. Mijakovic I. (2008). Phosphoproteome Analysis of *E. coli* Reveals Evolutionary Conservation of Bacterial Ser/Thr/Tyr Phosphorylation. Mol. Cell Proteomics 7, 299–307. 10.1074/mcp.M700311-MCP200 17938405

[B19] Malerba F. Alberini G. Balagura G. Marchese F. Amadori E. Riva A. (2020). Genotype-phenotype Correlations in Patients with De Novo KCNQ2 Pathogenic Variants. Neurol. Genet. 6, e528. 10.1212/NXG.0000000000000528 33659638 PMC7803337

[B20] Marin O. Meggio F. Sarno S. Cesaro L. Pagano M. A. Pinna L. A. (1999). Tyrosine versus Serine/Threonine Phosphorylation by Protein Kinase Casein Kinase-2. J. Biol. Chem. 274, 29260–29265. 10.1074/jbc.274.41.29260 10506183

[B21] Meggio F. Pinna L. A. (2003). One‐thousand‐and‐one Substrates of Protein Kinase CK2? FASEB j. 17, 349–368. 10.1096/fj.02-0473rev 12631575

[B22] Nakashima M. Tohyama J. Nakagawa E. Watanabe Y. Siew C. n. G. Kwong C. S. (2019). Identification of De Novo CSNK2A1 and CSNK2B Variants in Cases of Global Developmental Delay with Seizures. J. Hum. Genet. 64, 313–322. 10.1038/s10038-018-0559-z 30655572

[B23] O'Shea J. P. Chou M. F. Quader S. A. Ryan J. K. Church G. M. Schwartz D. (2013). pLogo: a Probabilistic Approach to Visualizing Sequence Motifs. Nat. Methods 10, 1211–1212. 10.1038/nmeth.2646 24097270

[B24] Okur V. Cho M. T. Henderson L. Retterer K. Schneider M. Sattler S. (2016). De Novo mutations in CSNK2A1 Are Associated with Neurodevelopmental Abnormalities and Dysmorphic Features. Hum. Genet. 135, 699–705. 10.1007/s00439-016-1661-y 27048600

[B25] Owen C. I. Bowden R. Parker M. J. Patterson J. Patterson J. Price S. (2018). Extending the Phenotype Associated with the CSNK2A1‐ Related Okur-Chung Syndrome-A Clinical Study of 11 Individuals. Am. J. Med. Genet. 176, 1108–1114. 10.1002/ajmg.a.38610 29383814

[B26] Perez-Riverol Y. Csordas A. Bai J. Bernal-Llinares M. Hewapathirana S. Kundu D. J. (2019). The PRIDE Database and Related Tools and Resources in 2019: Improving Support for Quantification Data. Nucleic Acids Res. 47, D442–D450. 10.1093/nar/gky1106 30395289 PMC6323896

[B27] Pinna L. A. (1990). Casein Kinase 2: An 'eminence Grise' in Cellular Regulation? Biochim. Biophys. Acta (Bba) - Mol. Cel Res. 1054, 267–284. 10.1016/0167-4889(90)90098-X 2207178

[B28] Pinna L. A. (2003). The Raison D'Être of Constitutively Active Protein Kinases: The Lesson of CK2. Acc. Chem. Res. 36, 378–384. 10.1021/ar020164f 12809523

[B29] Sarno S. Vaglio P. Marin O. Issinger O.-G. Ruffato K. Pinna L. A. (1997). Mutational Analysis of Residues Implicated in the Interaction between Protein Kinase CK2 and Peptide Substrates. Biochemistry 36, 11717–11724. 10.1021/bi9705772 9305961

[B30] Sarno S. Vaglio P. Meggio F. Issinger O.-G. Pinna L. A. (1996). Protein Kinase CK2 Mutants Defective in Substrate Recognition. J. Biol. Chem. 271, 10595–10601. 10.1074/jbc.271.18.10595 8631861

[B31] Schwartz D. Chou M. F. Church G. M. (2009). Predicting Protein Post-translational Modifications Using Meta-Analysis of Proteome Scale Data Sets. Mol. Cell Proteomics 8, 365–379. 10.1074/mcp.M800332-MCP200 18974045 PMC2634583

[B32] Soares N. C. Spät P. Krug K. Macek B. (2013). Global Dynamics of the *Escherichia coli* Proteome and Phosphoproteome during Growth in Minimal Medium. J. Proteome Res. 12, 2611–2621. 10.1021/pr3011843 23590516

[B33] Vilk G. Weber J. E. Turowec J. P. Duncan J. S. Wu C. Derksen D. R. (2008). Protein Kinase CK2 Catalyzes Tyrosine Phosphorylation in Mammalian Cells. Cell Signal. 20, 1942–1951. 10.1016/j.cellsig.2008.07.002 18662771

[B34] Walker C. Wang Y. Olivieri C. Karamafrooz A. Casby J. Bathon K. (2019). Cushing's Syndrome Driver Mutation Disrupts Protein Kinase A Allosteric Network, Altering Both Regulation and Substrate Specificity. Sci. Adv. 5, eaaw9298. 10.1126/sciadv.aaw9298 31489371 PMC6713507

[B35] Xu M. Cooper E. C. (2015). An Ankyrin-G N-Terminal Gate and Protein Kinase CK2 Dually Regulate Binding of Voltage-Gated Sodium and KCNQ2/3 Potassium Channels. J. Biol. Chem. 290, 16619–16632. 10.1074/jbc.M115.638932 25998125 PMC4505415

