# Supplementary material for: The Okur-Chung Neurodevelopmental Syndrome Mutation CK2K198R Leads to a Rewiring of Kinase Specificity
Source: Front Mol Biosci. 2022 Apr 19;9:850661. doi: 10.3389/fmolb.2022.850661 (PMC9062000; doi:10.3389/fmolb.2022.850661)
Supplement: Supplementary file 1 [file DataSheet1.PDF]

Supplementary Material

1.1 Supplementary Figures

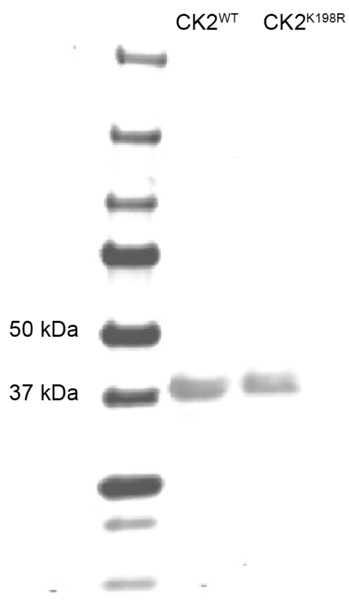

Supplementary Figure 1. $CK2^{WT}$ and $CK2^{K198R}$ are expressed at similar levels in *E. coli*.

Western blot showing expression levels of $CK2^{WT}$ and $CK2^{K198R}$. For both $CK2^{WT}$ and $CK2^{K198R}$ 25ug of lysate for (as determined by BCA assay concentrations) separated with SDS-PAGE and transferred to a PVDF membrane. The primary antibody used was Anti-CSNK2A1 antibody (Abcam ab10466), and the secondary antibody used was IRDye® 680RD Donkey anti-Rabbit IgG at a 1/5000 dilution.
